# Genome content reorganization in the non-model ciliate *Chilodonella uncinata*: insights into nuclear architecture, DNA content, and chromosome fragmentation during macronuclear development

**DOI:** 10.1128/msphere.00075-25

**Published:** 2025-05-09

**Authors:** Ragib Ahsan, Xyrus X. Maurer-Alcalá, Laura A. Katz

**Affiliations:** 1 Program in Organismic and Evolutionary Biology, University of Massachusetts Amherst 14707, Amherst, Massachusetts, USA; 2 Department of Biological Sciences, Smith College 6089, Northampton, Massachusetts, USA; 3 Division of Invertebrate Zoology and Institute for Comparative Genomics, American Museum of Natural History 5963, New York, New York, USA; Cleveland State University, Cleveland, Ohio, USA

**Keywords:** ciliate, genes, DNA content, chromosomes, genome dynamics, DAPI, telomere, FISH, confocal imaging, macronuclei, nuclear cycle, single-celled microorganisms

## Abstract

Ciliates are a model lineage for studies of genome architecture given their unusual genome structures. All ciliates have both somatic macronuclei (MAC) and germline micronuclei (MIC), both of which develop from a zygotic nucleus following sex (i.e., conjugation). Nuclear developmental stages are not well documented among non-model ciliates, including *Chilodonella uncinata* (class Phyllopharyngea), the focus of our work. Here, we characterize nuclear architecture and genome dynamics in *C. uncinata* by combining 4′,6-diamidino-2-phenylindole (DAPI) staining and fluorescence *in situ* hybridization (FISH) techniques with confocal microscopy. We developed a telomere probe for staining, which alongside DAPI allows for the identification of fragmented somatic chromosomes among the total DNA in the nuclei. We quantify both total DNA and telomere-bound signals from more than 250 nuclei sampled from 116 individual cells, and analyze changes in DNA content and nuclear architecture across *Chilodonella*’s nuclear life cycle. Specifically, we find that MAC developmental stages in the ciliate *C. uncinata* are different from those reported from other ciliate species. These data provide insights into nuclear dynamics during development and enrich our understanding of genome evolution in non-model ciliates.

**IMPORTANCE:**

Ciliates are a clade of diverse single-celled eukaryotic microorganisms that contain at least one somatic macronucleus (MAC) and germline micronucleus (MIC) within each cell/organism. Ciliates rely on complex genome rearrangements to generate somatic genomes from a zygotic nucleus. However, the development of somatic nuclei has only been documented for a few model ciliate genera, including *Paramecium*, *Tetrahymena*, and *Oxytricha*. Here, we study the MAC developmental process in the non-model ciliate, *C. uncinata*. We analyze both total DNA and the generation of gene-sized somatic chromosomes using a laser scanning confocal microscope to describe *C. uncinata*’s nuclear life cycle. We show that DNA content changes dramatically during their life cycle and in a manner that differs from previous studies on model ciliates. Our study expands knowledge of genome dynamics in ciliates and among eukaryotes more broadly.

## INTRODUCTION

Ciliates are an approximately 1-billion-year-old clade of diverse eukaryotic microorganisms (1–4). One of the key characteristics of ciliates is the presence of dimorphic nuclei within a single individual, where ciliates possess at least one somatic macronucleus (MAC) and at least one germline micronucleus (MIC) (5, 6). Somatic macronuclei are highly polyploid with active gene expression throughout the life cycle (7–10), whereas the germline micronucleus is diploid and remains quiescent during asexual growth (6). Germline micronuclei generate gametic nuclei through meiosis, which are exchanged during conjugation (8, 11–16). The number, shape, and structure of MACs and MICs vary among species in ciliates (5).

Ciliates have unusual nuclear structures and complex life cycles in which they alternate between asexual division and sexual conjugation. During asexual reproduction, most ciliates reproduce by binary fission during which micronuclei divide by mitosis and polyploid macronuclei divide by amitosis (17). Sex in ciliates occurs through conjugation, where germline micronuclei produce haploid products through meiosis, which are exchanged between cells (16–20), and then fuse to form a zygotic nucleus. This zygotic nucleus divides mitotically, with one daughter nucleus developing into a new somatic macronucleus and the other becoming a new germline micronucleus (5). Although nuclear structure and genome dynamics are well explored in several model ciliates (*Tetrahymena*, *Oxytricha*, and *Paramecium*) (8, 21–27), the extent of how well these processes reflect the bulk of ciliate diversity remains poorly understood (28–31).

Here, we focus on *Chilodonella* (class Phyllopharyngea), as it is both cultivable and among non-model ciliates whose nuclear architecture is yet to be fully explored. *Chilodonella*’s somatic nuclear architecture is quite distinct, harboring dense DNA-rich sphere-like heteromeric structures (32) that surround a DNA-poor center. The density and characteristics of these DNA-rich “spheres” vary across *Chilodonella*’s nuclear life cycle (17, 32).

There are some prior studies that focused on describing *Chilodonella*’s MAC development using light and electron microscopy (33–35), its life cycle (17), and aspects of its genome biology and development (30, 32). Here, we propose a revised life cycle for the non-model freshwater ciliate *Chilodonella uncinata* based on modern fluorescence-based descriptions of the macronuclear developmental stages. By combining 4′,6-diamidino-2-phenylindole (DAPI) staining and fluorescence *in situ* hybridization (FISH) targeting the telomere sequences of this species, we are able to explore changes in the total DNA content and somatic chromosome maturation across developmental stages. This is largely feasible as the somatic nucleus of *C. uncinata* contains thousands of gene-sized telomere-containing “chromosomes” that are generated from a much smaller number of zygotic chromosomes (6, 36, 37). Hence, DAPI stains both germline and somatic nuclei while telomere-FISH specifically targets somatic chromosomes. We also evaluate the variation in nuclear architecture during conjugation and relate nuclear data to estimates of cell size. Overall, this study contributes to our understanding of nuclear life cycles and particularly in the development of somatic macronuclei in *C. uncinata*.

## MATERIALS AND METHODS

### Cell culture and maintenance

Cultures of *Chilodonella uncinata* (strain ATCC PRA-257) were originally isolated from a sample in Poland in 2014 and were maintained in Volvic water (bottled Volvic natural spring water) with autoclaved rice grains to support bacterial (i.e., prey) growth. Cultures of *C. uncinata* were maintained in 6-well plates, transferring 200 µL of cells into new wells with some Volvic water every 3–5 days. All cultures were maintained at room temperature and in the dark. We used a brightfield microscope (Olympus CKX31) to maintain the cultures and isolate cells for FISH experiments.

### Cell isolation for the experiment and fixation

Cells isolated from the 6-well plate wells were transferred to Superfrost slides (Fisherbrand Superfrost Plus Microscope Slides; Catalog No. 22-037-246) using a 200 µL micropipette. Before placing the isolated cells on the superfrost slides, a square-shaped boundary was drawn on the slides using a hydrophobic pen (Cole-Parmer Hydrophobic Barrier PAP Pen; Catalog No. NC1882459). Cells were left on the Superfrost slides at room temperature for 20 minutes. Afterward, a solution of 4% paraformaldehyde (PFA, product ID: J19943-K2, lot #210699) in phosphate-buffered saline (PBS) (product code: 1003127976, lot #SLCH0992) was added at a final concentration of 2% and incubated at room temperature for 20 minutes. The majority of the liquid was then drawn off the slides, followed by three washes with 1× PBS.

### Fluorescence *in situ* hybridization

We performed FISH to localize the gene-sized chromosomes in the macronuclei in *Chilodonella uncinata*. We designed the oligonucleotide telomere probe, labeled with Alexa Fluor 488 fluorescent dye at the 5’, using the direct telomeric repeats (C_4_AAA_3_)_3_, which was previously determined by cloning gene-sized somatic chromosomes (38). The use of this probe enables us to estimate the relative copy number of gene-sized somatic (MAC) chromosomes throughout the life cycle of *C. uncinata*.

Following fixation and washing, cells were permeabilized using 0.5% Triton X-100 for 20 minutes at room temperature, then washed twice with 1× PBS and once with 4× saline-sodium citrate (SSC). Cells were equilibrated in a pre-hybridization buffer (50% formamide, 2× SSC, and nuclease-free water [NFW]) for 30 minutes at room temperature. The hybridization buffer was prepared by mixing 10 µM telomere probe, 50% formamide, 4× SSC solution, and NFW. This hybridization mix was denatured at 98°C for 5 minutes and was snap cooled on ice. Hybridization buffer (20 µL) was added to the cells, which were incubated at 75°C for 5 minutes, then overnight at 37°C. The next day, slides were washed with 2× SSC for 15 minutes at 37°C, then 1× SSC for 15 minutes at 37°C in a water bath, with a final wash with 1× SSC at room temperature for 15 minutes. Total DNA counterstaining was performed using 0.001 µg/mL DAPI for 2 minutes. Afterward, cells were washed once in 1× PBS solution, and a drop of Slow Fade Gold was applied on the cells before covering the cells with a cover glass and sealing it with nail polish.

### Microscopy and imaging

Stained cells were assessed using a Leica TCS SP5 laser-scanning confocal microscope (Leica, Mannheim, Germany). Images were captured with a 63× oil immersion objective. Total DNA (DAPI) was excited with a UV laser at 405 nm, differential interference contrast (DIC) images were captured with an argon laser (488 nm), while the Alexa Fluor 488-conjugated telomere probe was excited by a wavelength of 488 nm. All images were captured at a resolution of 1,024 × 1,024, an acquisition speed of 200 Hz, and a line averaging of 16. Images were sequentially scanned with the aim of generating red, green, and blue (RGB) color images with an 8-bit depth configuration. We only report cells with good overall morphology, passing over cells that were folded, crushed, or otherwise suboptimal and likely representing preparation-induced artifacts. z-stacks images were taken with an acquisition speed of 700 Hz (although we initially recorded some z-stacks with an acquisition speed of 400 Hz), a line averaging of 4, and a step size of 0.13 µm. Each z-stacks takes roughly 45–75 minutes, so the images included here represent ~116 hours for all the cells recorded in this study. We adjusted the smart gain and smart offset to improve image quality.

### Image analysis

Cell/nuclear volume and total fluorescence intensity were quantified using the “Nikon NIS Element” image analysis software (Nikon, Tokyo, Japan). We manually set points (or outlines) to capture cell size (length × width in micrometers) and nuclear diameter (by drawing a circle around the nucleus and measuring the diameter in micrometers) using the measurement tools in the “Nikon NIS Element” software to calculate their respective radius (in micrometers) and volume (in cubic micrometers). The mean intensity of all nuclei was calculated using the “ROI (region of interest)” tool in the “Nikon NIS Element” software. Using the ROI tool, we drew a polygonal or circular line surrounding the nucleus, returning the mean intensity using the “ROI” statistics. Total intensity was calculated by multiplying the nuclear volumes (cubic micrometers) with the mean intensity per pixel.

## RESULTS

**Fig 1:**
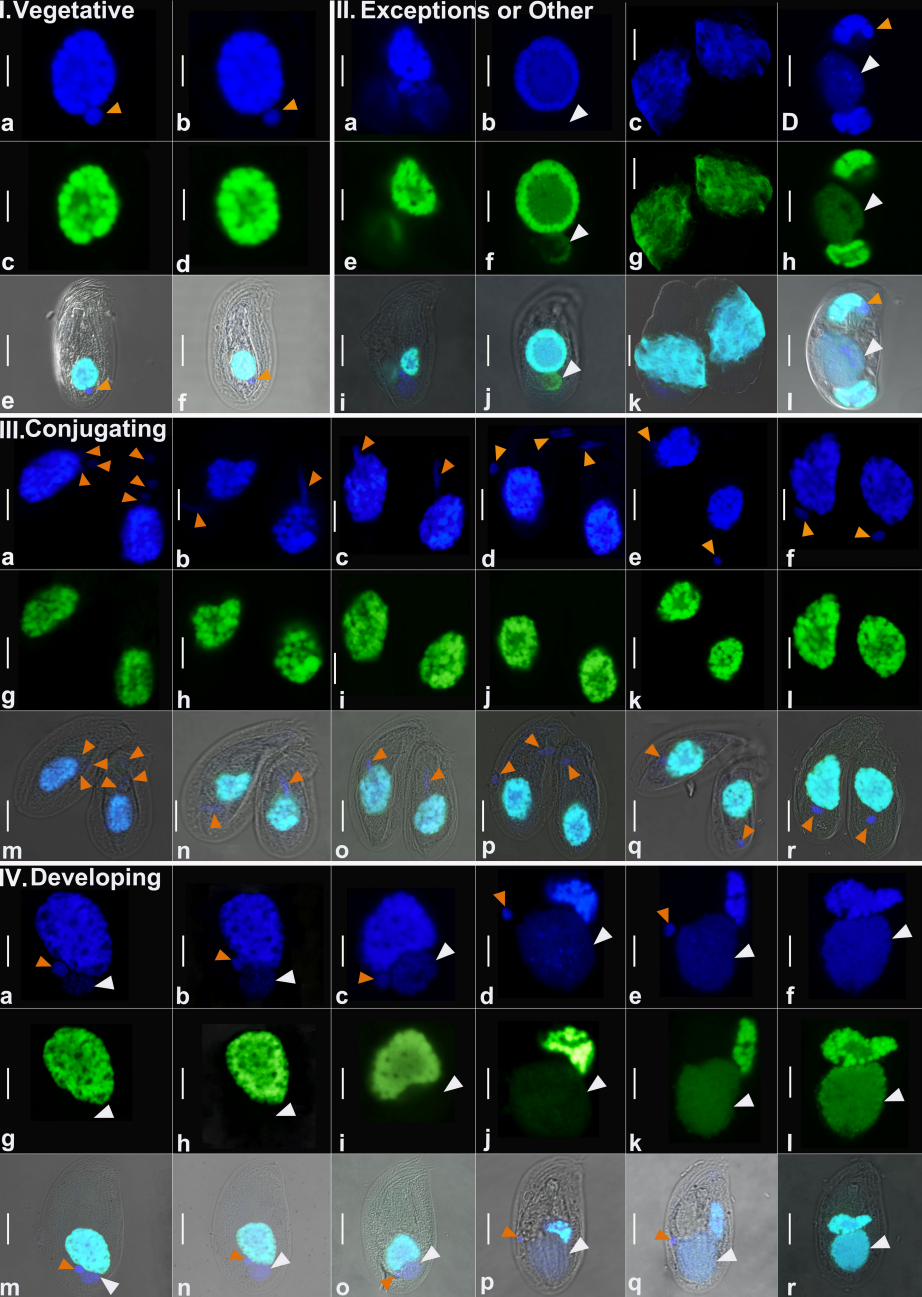
Representative images from each categorized developmental stage, DAPI-stained (blue) and Telomere-stained (green) nuclei, and overlay for each of *C. uncinata* cell. (I) Representative cells and nuclei from vegetative stage (a–f). (II) All the exceptional forms of nuclei that were captured during the study but not categorized. (III) Representative conjugating cells and their nuclei ( a–r). (IV) Representative developing cells and their nuclei; panels a–c, g–i, and m–o represent the early developing stage; panels d–f, j–l, and p–r represent the late developing stage. The rows (I) e and f, (II) i–l, (III) m–r, and (IV) m–r represent cells that contain both the nuclei within the same individual. We adjusted the brightness and contrast of these overlay images to enhance visualization of all overall cells. All raw images for this study are accessible as described in our Data Availability statement. Orange arrowheads indicate the MICs, and white arrowheads indicate the developing MACs. MICs, micronuclei; MACs, macronuclei. Scale bar = 5 µm.

**Fig 2:**
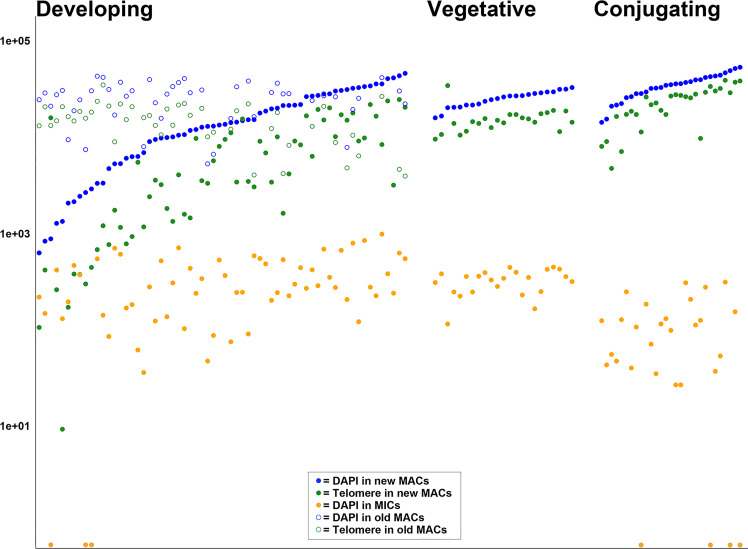
Different stages and total intensity during the macronuclear development in *C. uncinata* of all categorized cells. The *x*-axis represents the different stages during the macronuclear development, and the *y*-axis (in log scale) represents the total intensity (i.e. DAPI or telomere probe). Cells are categorized by “Developing“ (including early and late developing stages), “Vegetative,” and “Conjugating“ stages from left to right on the *x*-axis. Solid blue circles are the newly developing MACs DNA data, and solid green circles are the newly developing MACs telomere data, respectively. Open blue circles are the old MACs DNA data, while open green circles are the old MACs telomere data, respectively, during early and late development. Solid orange circles indicate the MICs data on the plot. MICs, micronuclei; MACs, macronuclei.

**Fig 3:**
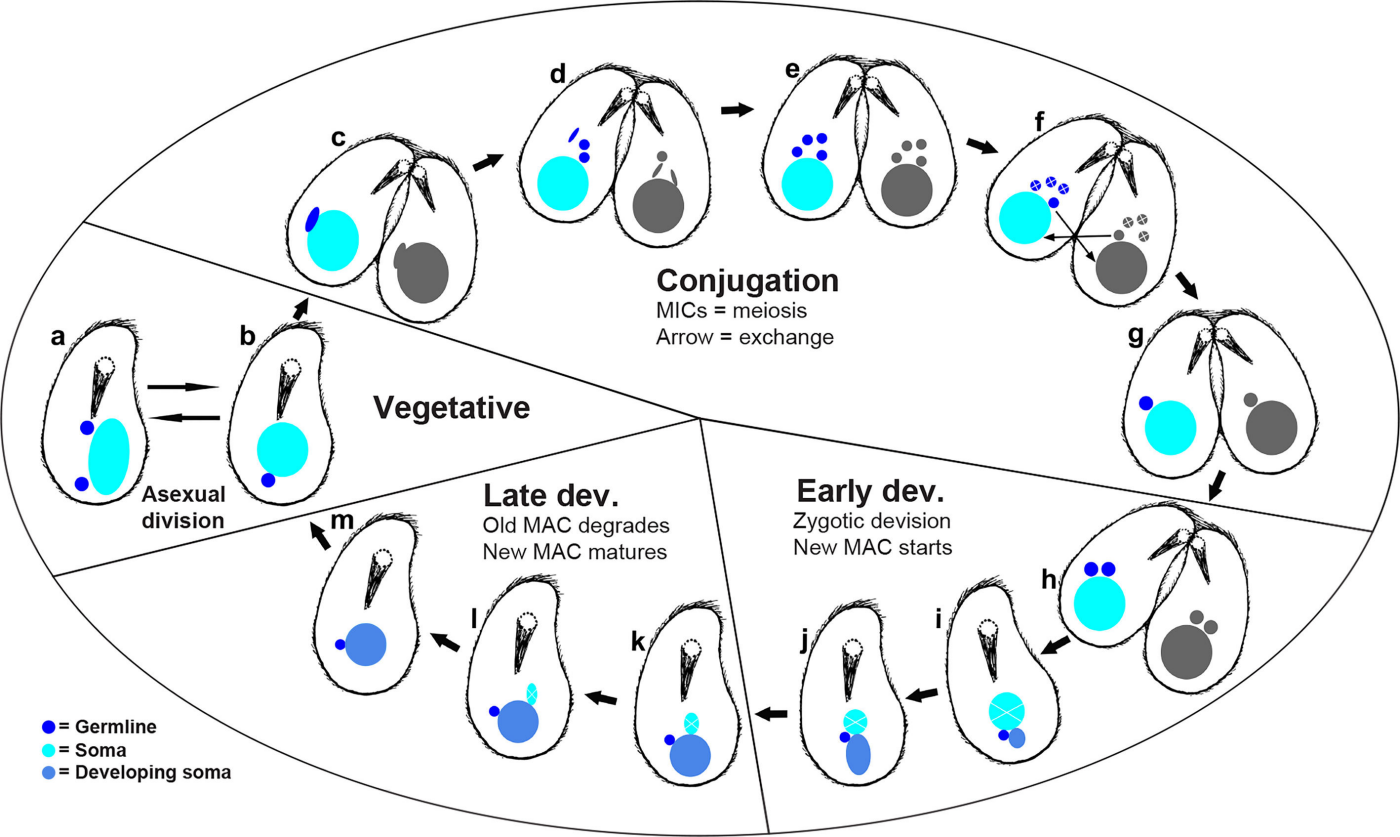
Inferred life cycle of *Chilodonella uncinata* illustrated by cartoons based on images from Fig. 1 and Fig. S1 to S8. (a) MAC amitosis (asexual division). (b) Vegetative cells and nuclei. (c) Meiosis-I. (d–f) Conjugation, meiosis-II of MICs, and exchange of haploid MICs. (g and h) Zygotic nuclei and mitotic division of zygotic nuclei. (i and j) Early development of the new MAC occurs as the parental MAC shrinks (Fig. 1), yet DNA content remains relatively stable in the parental MAC at this stage (Fig. 2). (k–m) Late development of the new MACs occurs as the parental MAC disappears. MICs, germline micronuclei; MACs, somatic macronuclei.

We used laser-scanning confocal microscopy to characterize nuclear features from more than 250 nuclei sampled from 116 individuals, which were stained with both DAPI and our telomere-specific probe (Tables S1 and S2; Table 1). We evaluated cell and nuclear size, volume, total DNA (as estimated by DAPI), and amount of gene-sized chromosomes using a telomere-binding FISH probe (Table 1; Table S2; Fig. 1 and 2). In total, we captured 23 vegetative, 29 conjugating, and 64 developing (early and late developing) cells, which allowed us to infer the life cycle stages (Fig. 3) of *C. uncinata* with greater detail. Comparing across life cycle stages, we found that the conjugating cells tend to be smaller in size, averaging ~31 µm in length, compared to vegetative (~40 µm in length) and developing (~35 µm in length) (Fig. S9). We also found a number of cells that did not easily fit in one of these three categories, and we include them as oddities, which may represent either preparation artifacts or rare events (Fig. 1, panel II. a–l).

**TABLE 1:** Image analysis summary table of *Chilodonella uncinata* cells at different stages during their macronuclear development shows variability in their total DNA and telomere content during their MAC development^*a*^

Categorized dev stages	No. of nuclei quantified	Avg cell vol (µm^3^)	Avg nuc vol (µm^3^)	MAC–cell ratio	Avg TI (DNA)	Avg TI (Telo)	Avg DAPI vs Telo ratio
Vegetative	23	8171.7	168.8	0.27	24,716	14,122	0.63
Early development	40	7,236.48	142.4	0.20	13,758	6,725	0.83
Late development	24	1,1201.01	316.9	0.40	20,566	7,433	0.32
Conjugating	29	5,914.46	225.4	0.48	33,788	22,223	0.64

^*a*^ Avg, average; TI, total intensity; vol, volume; dev, developing; MAC, macronucleus.

### Vegetative cells

We defined 23 vegetative cells analyzed in this study (Fig. S1; Table 1; Tables S1 and S2) as those containing a large “typical” heteromeric macronucleus (e.g., with densely stained material surrounding a DNA-poor center) with a smaller germline micronucleus below the MAC (Fig. 1Ia–f; Fig. S1 and S8). The vegetative macronuclei stain robustly with both DAPI (blue, total DNA) and the telomere probe (green, MAC chromosomes) (Fig. 2), consistent with the endoreplication of gene-sized chromosomes as seen in *C. uncinata* (32) and the congeners *Chilodonella cucullulus* and *Chilodonella steini* (39, 40). In contrast, no obvious fluorescence signal for the telomere probe was detected in the germline micronuclei, which are predicted to have only a small number of chromosomes and hence few telomeres (Fig. 1Ia–f; Fig. S1 and S2).

We observed a consistent ratio between total DNA (i.e., DAPI) and telomere signal in the vegetative cells, with only one cell possessing more telomere signal than DAPI (Fig. S1 and S2; Table S1). The consistent ratio between estimates of the total DNA content and chromosome numbers (estimated by telomere-based fluorescence, Fig. S11) coupled with the near doubling of both based on the relative fluorescence units (from ~16,000 to 32,000 and from ~9,000 to 18,000 for DAPI and telomere probe, respectively; Table S1; here, we exclude an aberrant cell that appeared to have three times as much telomere signal than expected) indicates that these vegetative cells are likely cycling through asexual vegetative growth stages, including preparation for the amitotic division of macronuclei. We did not capture mitosis of micronuclei among these 23 vegetative cells.

### Conjugation

We collected data from a total of 29 conjugating cells, which we identified as pairs of cells joined at their oral apertures (Fig. 1IIIa–r; Fig. S3; Table 1; Table S2). To allow comparisons across life history stages, we choose to measure only one cell, arbitrarily choosing the cell on the right side of all conjugating pairs (Fig. 1IIIa–r; Fig. S3). Across all the conjugating cells we analyzed, we were able to capture their meiotic events (both meiosis-I and meiosis-II), plus exchanging of nuclei (Fig. 3c through f; Fig. S4). The ratio between the total DNA (in blue) and telomeres (in green) varies more across the conjugating cells than the vegetative cells (Fig. 2; Fig. S11 and S12). In addition, we note that these cells have a similar total DNA content as vegetative cells (Fig. 2) despite their smaller size (Fig. S3 and S9).

### Development of somatic macronuclei

Given our interest in nuclear architecture, the bulk of our analyses focused on individuals in various stages of development. After measuring a total of 64 cells determined to be developing based on the presence of both a new and an old somatic macronucleus, we inferred developmental stages (Fig. 1IVa–r; Fig. S5 and S7). We categorized developing cells into two broad subcategories: (i) early development as those stages that still contain prominent old MACs and only weakly stained new MACs, and (ii) late development as stages with prominent new MACs. Using these criteria, we categorized 40 cells being in early development and 24 in late development stages (Table 1; Table S2). We report on DNA and telomere staining of both the “old MAC” (i.e., the one degrading over time) and the “new MAC” (i.e., the anlagen, or newly developing nucleus) (Fig. 1IVa–c; Fig. S5 and S6), and provide original images and z-stacks in our shared Google Drive folder (see Data Availability statement).

During development, the ratio between the total DNA and estimated telomere content varies (Fig. S11 and S12), although both increase gradually between early and late developmental stages. By comparing the total DNA and amount of gene-size chromosomes between the newly developing macronucleus and the “old” macronucleus (Fig. S10), we observe that the DNA content in the old MACs does not decrease as the new MAC develops (Fig. S10B), although there is a decline in telomere signal (Fig. S10C). This suggests that *C. uncinata* may not be synchronously recycling old material as it generates a new macronucleus following conjugation (see below).

Based on our detailed observations of ~250 nuclei from >100 *C*. *uncinata* individuals, we propose a revised nuclear life cycle (Fig. 3; Table S1). During conjugation, micronuclei become elongated and increase in their number, which we interpret as meiosis-I (Fig. 3a). Next, we see additional elongated stages with up to four micronuclei per cell, consistent with meiosis-II. We inferred that three of these haploid nuclei degrade, and the remaining haploid nucleui in each individual undergo mitosis before being exchanged with their partner cell (Fig. 3d–f) and undergoing nuclear fusion (karyogamy). The resulting zygotic nucleus undergoes mitotic division; one of the daughter nuclei becomes the new MAC as the other is the new MIC in the mature cell (Fig. 3g and h).

During *Chilodonella*’s early development, the old MAC retains its “typical” morphology while the newly developing MACs appear attached to them (Fig. 1IV; Fig. 3i through m). We found a consistent positioning of the old and new (developing) MACs during the early and late developing stages: the old MACs tend to be on “top” (toward the anterior portion of the cell), and the newly developing (new) MACs position on the “bottom” (toward the posterior portion of the cell—under the old MACs) of the old MACs (Fig. 3i through m; Fig. S5). During the late developmental stage, the old MACs lose their typical morphology and degrade as the new MACs develop with the new MIC attached or almost attached (Fig. 1IVd–f, j–l, and p–r; Fig. 3i through m). After going through all of these stages, they return to their vegetative stage (Fig. 3b; Fig. S1). We recorded a few cases (although we saw many) where the MAC was dividing into two roughly equal parts, which we infer as asexual division (Fig. 3a).

### Exceptions

While scanning thousands of cells under the microscope, we found a few cells with nuclear stages that seemed unusual. One cell contains chromosomes in the newly developing MAC but little or no DNA in the same developing MAC (Fig. 1IIb, f, and j), two individuals that had three nodules of MACs with different patterns of its position (Fig. 1IIa, e, and i and d, h, and l ), and one individual has very densely spread out DNA (Fig. 1IIc, g, and k). It is unclear whether these exceptions represent unknown plasticity or dead ends in the life cycle of *C. uncinata*.

## DISCUSSION

Here, we characterize nuclear events in the life cycle of a non-model ciliate, *Chilodonella uncinata*, using FISH and laser-scanning confocal microscopy. We investigate total DNA with DAPI staining as well as fluorescence-based estimates of the abundance and distribution of *Chilodonella*’s gene-sized macronuclear chromosomes (36, 41–43) with a telomere-specific FISH probe. We report patterns of macronuclear development that differ from those previously described for diverse model lineages and even for the congeners *C. cucullulus* and *C. steini* (39, 40). We also find unexpected variability in macronuclear DNA content during conjugation and present an updated life cycle for this species that extends from a previous study in our laboratory (17).

Some aspects of *C. uncinata*’s nuclear processes are similar to the model ciliates (*Tetrahymena*, *Paramecium*, *Oxytricha*), as all lineages have distinct germline and somatic nuclei, with somatic nuclei developing from a zygotic nucleus following conjugation. Yet other aspects are unique to *C. uncinata*: (i) consistent with previous work (17, 39, 40), DNA is organized into clear DNA rich and poor regions (heteromeric) in the somatic nucleus, (ii) content in the heteromeric somatic macronucleus doubles during amitotic division, and (iii) the ratio of total DNA to gene-sized chromosome abundance remains relatively stable (Fig. 1Ia–f; Fig. S1 and S2; Fig. 2). In other ciliates with extensively fragmented genomes (i.e., possessing gene-sized chromosomes), the ratio of the total DNA content to gene-sized chromosomes has not been reported by microscopy.

In contrast to the relative consistency in vegetative stages (Fig. 2), the ratio of total DNA to gene-sized chromosomes in *C. uncinata* is much more variable during conjugation (Fig. S12), including when meiotic divisions of the germline micronucleus are occurring (Fig. 3c through h). It is unclear why this variability occurs, although it may be in response to the state of the cell and/or the available resources, which requires further study across a broader sampling of ciliates, including those with atypical nuclear architectures (e.g., *Chilodonella*). During development, *Stylonychia* may “recycle” nucleotides from the hyperpolyploid parental or old somatic nucleus to offset the energetic cost of DNA amplification during the development of the new somatic nucleus. However, we observe no clear evidence of such recycling in *Chilodonella*. Rather, the newly developing MAC’s DNA content increases before the apparent degradation of the parental MAC (Fig. 2; Fig. S6 and S8), as measured by relative fluorescence units (21, 44–47). The fate of the nucleotides in the parental (old) macronucleus in *C. uncinata* remains unclear, as these nucleotides may be broken down or used in subsequent life history stages (e.g., the first/subsequent asexual divisions).

*Chilodonella uncinata*’s MAC development differs markedly from other ciliates with gene-sized chromosomes. For example, spirotrich ciliates like *Stylonychia, Oxytricha,* and *Euplotes* go through three clear development stages: (i) an initial amplification stage of the entire genome, (ii) a DNA-poor stage (result of massive DNA elimination), and (iii) a final amplification of mature gene-sized chromosomes (45, 48). We observe no evidence of a distinct DNA elimination stage in *C. uncinata*. Instead, there is a concerted increase in the amount of total DNA and abundance of gene-sized chromosomes throughout the development of the new MAC (Fig. 2). This remains consistent with a prior macronuclear development study of this species (35), which suggests that DNA elimination is likely ongoing throughout development, resemblant of *Paramecium*’s somatic development (6). Notably, *Chilodonella* is estimated to eliminate at least ~30–35% of its germline (49–51), compared to *Stylonychia* that eliminates >90% of its germline genome (21, 52), which may be a driver for nucleotide recycling in the latter species.

### Synthesis

We present an updated life cycle of *C. uncinata* that incorporates the data we collected by fluorescence microscopy (Fig. 3). Here, we show changes in the position of the meiotic products prior to conjugation, and of both the newly developing MAC and MIC during development. We observed changes in the MIC position during developmental events, with the MIC repositioning from the posterior region of the cell to being nestled between the old and developing somatic nuclei. Most studies of nuclear development in other ciliates report that the developing MAC is often positioned toward the posterior portion of the cell, which we also report in *C. uncinata* (23, 27, 44, 48, 53, 54). Together, these data from *Chilodonella uncinata* expand our knowledge of ciliate life cycles.

## Data Availability

All original images and z-stacks are uploaded in an accessible Google Drive folder (https://drive.google.com/drive/folders/1gpm2cU0kJOh9_UgIZFl8em8BUf8esYR3).

## SUPPLEMENTAL MATERIAL

Supplemental material msphere.00075-25-s0001.pdf Figure S1 to S12 and supplemental table captions.

Table S1 msphere.00075-25-s0002.xlsx Detailed information of all individual cells recorded in this study.

Table S2 msphere.00075-25-s0003.xlsx Mean, median, and SD values for Table 1.

ASM does not own the copyrights to Supplemental Material that may be linked to, or accessed through, an article. The authors have granted ASM a non-exclusive, world-wide license to publish the Supplemental Material files. Please contact the corresponding author directly for reuse.
