# Supplementary material for: Genome content reorganization in the non-model ciliate Chilodonella uncinata: insights into nuclear architecture, DNA content, and chromosome fragmentation during macronuclear development
Source: mSphere. 2025 May 9;10(6):e00075-25. doi: 10.1128/msphere.00075-25 (PMC12188737; doi:10.1128/msphere.00075-25)
Supplement: Supplemental material — Figure S1 to S12 and supplemental table captions. [file msphere.00075-25-s0001.pdf]

SUPPLEMENTARY MATERIALS

Supplementary figure legends:

Figure S1: All vegetative cells in this study (a-w) showing cells with DAPI and telomere overlay. Scale bar = 5μm.

Figure S2: All vegetative nuclei in this study (a.i-w.ii), showing all the DAPI (blue) and telomere (green) stained nuclei from vegetative cells. Scale bar = 2μm.

Figure S3: All conjugating cells of *Chilodonella uncinata* in this study (1-29), showing all cells with DAPI and telomere overlay. Scale bar = 5μm.

Figure S4: All conjugating cell nuclei in this study (1.i-29.ii), showing all the DAPI (blue) and telomere (green) stained nuclei from conjugating cells separately. Scale bar = 2μm.

Figure S5: All early developing cells of *Chilodonella uncinata* (1-40) showing DAPI and telomere stained overlays. Scale bar = 5μm.

Figure S6: All early developing cell nuclei i (1.i-40.ii) with DAPI (blue) and telomere (green) stained nuclei. Scale bar = 2μm.

Figure S7: All late developing cells of *Chilodonella uncinata* in this study(a-x), showing DAPI and telomere stained overlay. Scale bar = 5μm.

Figure S8: All late developing cell nuclei in this study (a.i-x.ii) showing all the DAPI (blue) and telomere (green) stained nuclei. Scale bar = 2μm.

Figure S9: Box plot of life stage vs. cell volume in for all *Chilodonella uncinata* cells study; conjugating cells have relatively smaller cell volume than the other two categories.

Figure S10: Plots to compare the total DNA in developing macronuclei (MAC) to the total telomere in developing MACs (A), to the total DNA in old MACs (B), and to the total telomere in old MACs (C). Dev.= Developing; MAC= Macronuclei.

Figure S11: Plots to compare the total intensity (TI) of DNA (DAPI) vs telomere (Tel) of all the stages. Comparison of total intensity between the DNA and telomere in (A) vegetative stage; (B) conjugating stage; (C-D) early & late developing stages; (E-F) early & late developing stages with O-MAC telomere. TI= Total Intensity; MAC= Macronuclei; O-MAC= Old Macronuclei.

Figure S12: Box plot of the comparison between life stages vs. ratio of the total fluorescence of telomere & DNA in this study showing developing cells have the relatively variable Tel TF/D TF ratio than the other life stages. Tel= Telomere; TF= Total Fluorescence; D= DAPI/DNA.

Supplementary table legends:

Table S1: Detailed information of all individual cells recorded in this study.

Table S2: Mean, median, and standard deviation (SD) values for Table 1.

Figure S1:

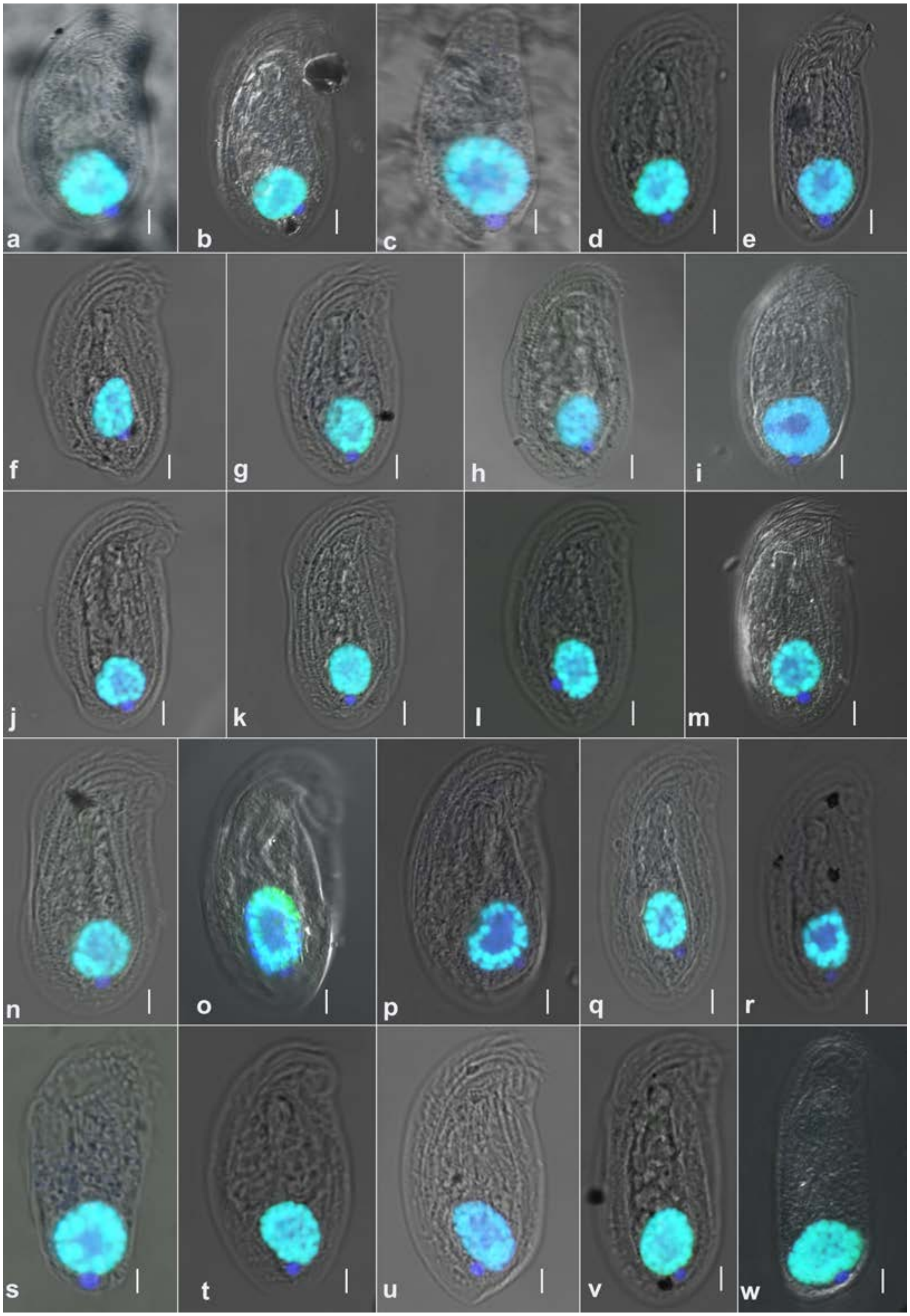

Figure S2:

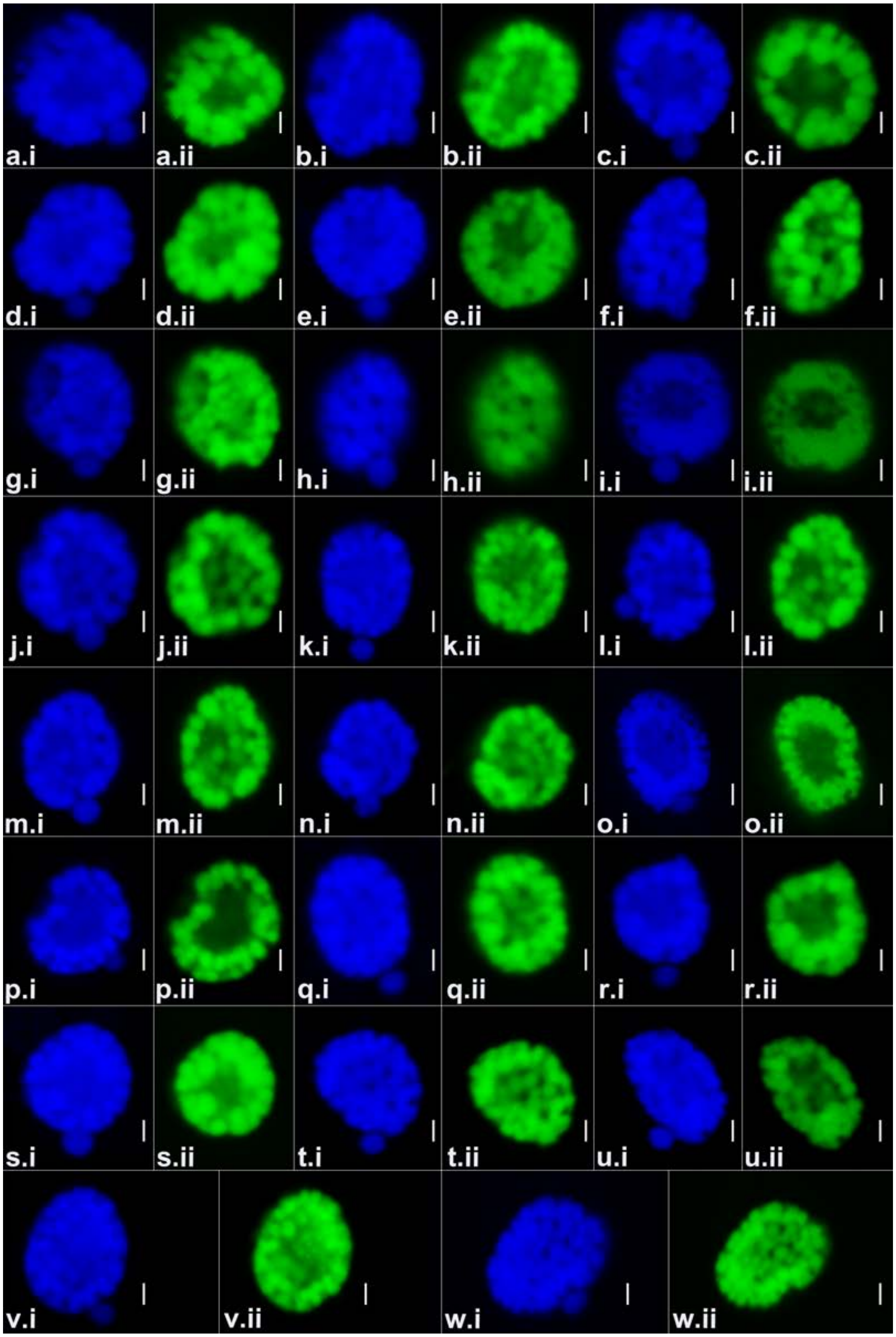
a.i
a.ii
b.i
b.ii
c.i
c.ii
d.i
d.ii
e.i
e.ii
f.i
f.ii
g.i
g.ii
h.i
h.ii
i.i
i.ii
j.i
j.ii
k.i
k.ii
l.i
l.ii
m.i
m.ii
n.i
n.ii
o.i
o.ii
p.i
p.ii
q.i
q.ii
r.i
r.ii
s.i
s.ii
t.i
t.ii
u.i
u.ii
v.i
v.ii
w.i
w.ii

Figure S3:

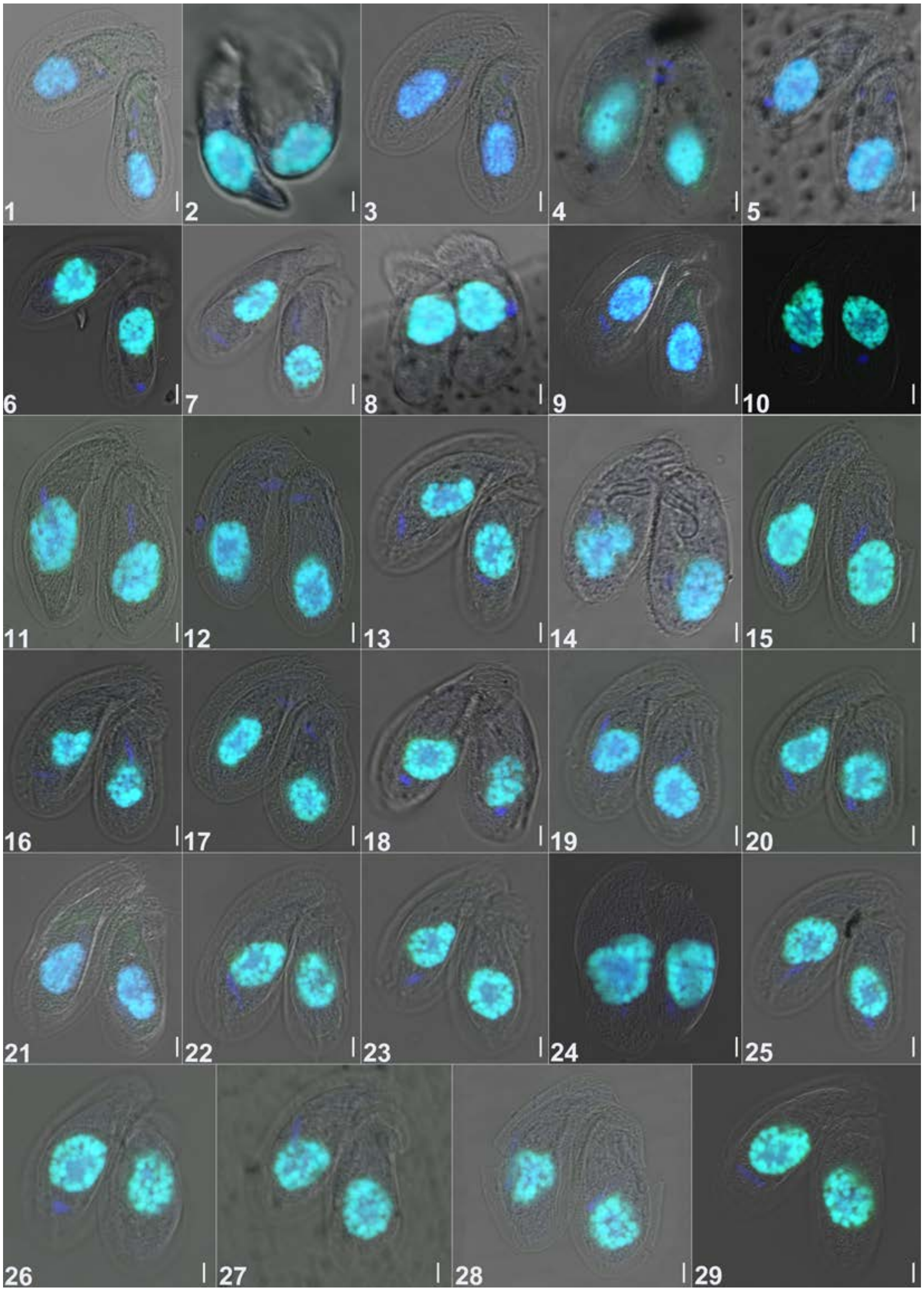

Figure S4:

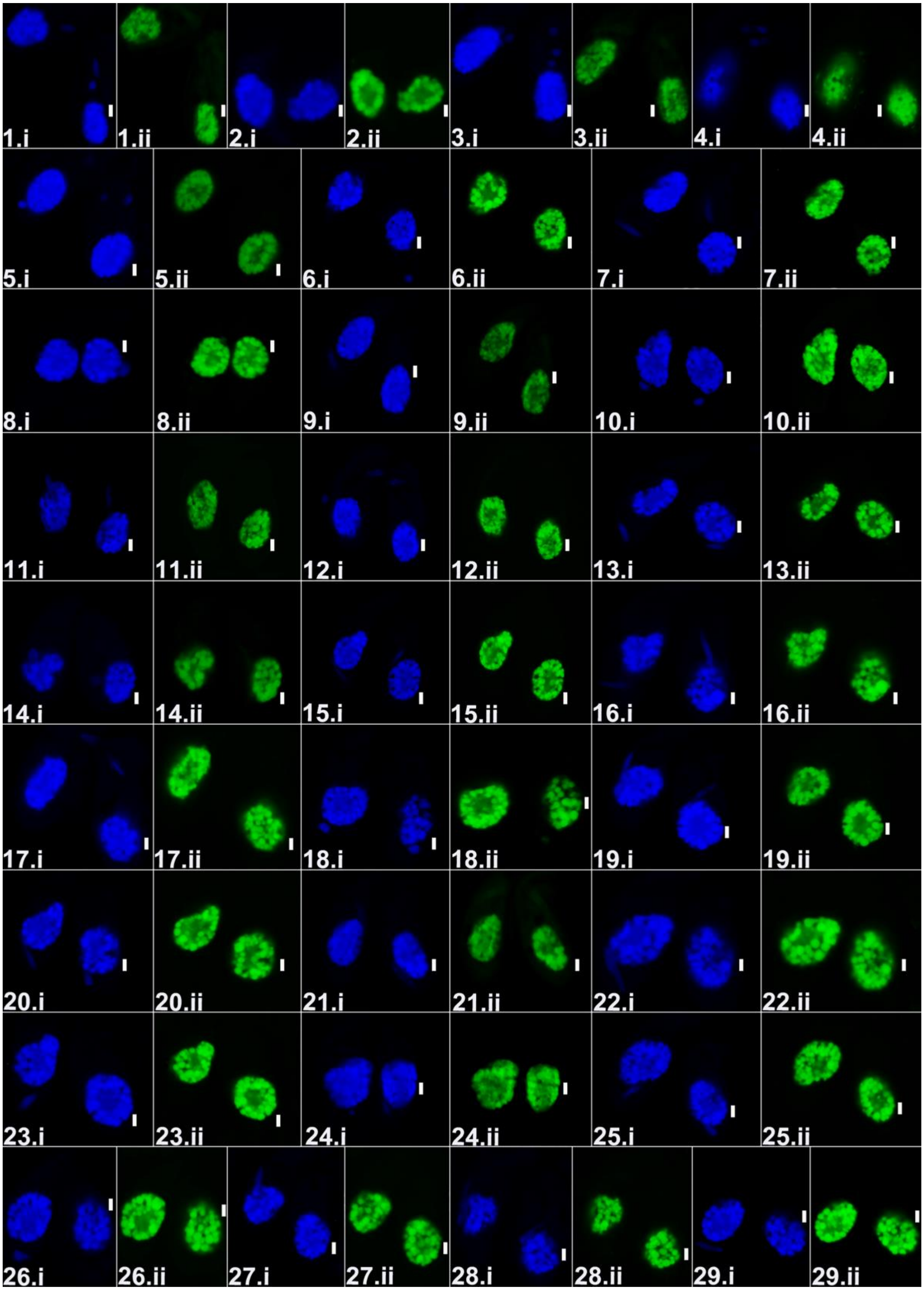
1.i
1.ii
2.i
2.ii
3.i
3.ii
4.i
4.ii
5.i
5.ii
6.i
6.ii
7.i
7.ii
8.i
8.ii
9.i
9.ii
10.i
10.ii
11.i
11.ii
12.i
12.ii
13.i
13.ii
14.i
14.ii
15.i
15.ii
16.i
16.ii
17.i
17.ii
18.i
18.ii
19.i
19.ii
20.i
20.ii
21.i
21.ii
22.i
22.ii
23.i
23.ii
24.i
24.ii
25.i
25.ii
26.i
26.ii
27.i
27.ii
28.i
28.ii
29.i
29.ii

Figure S5:

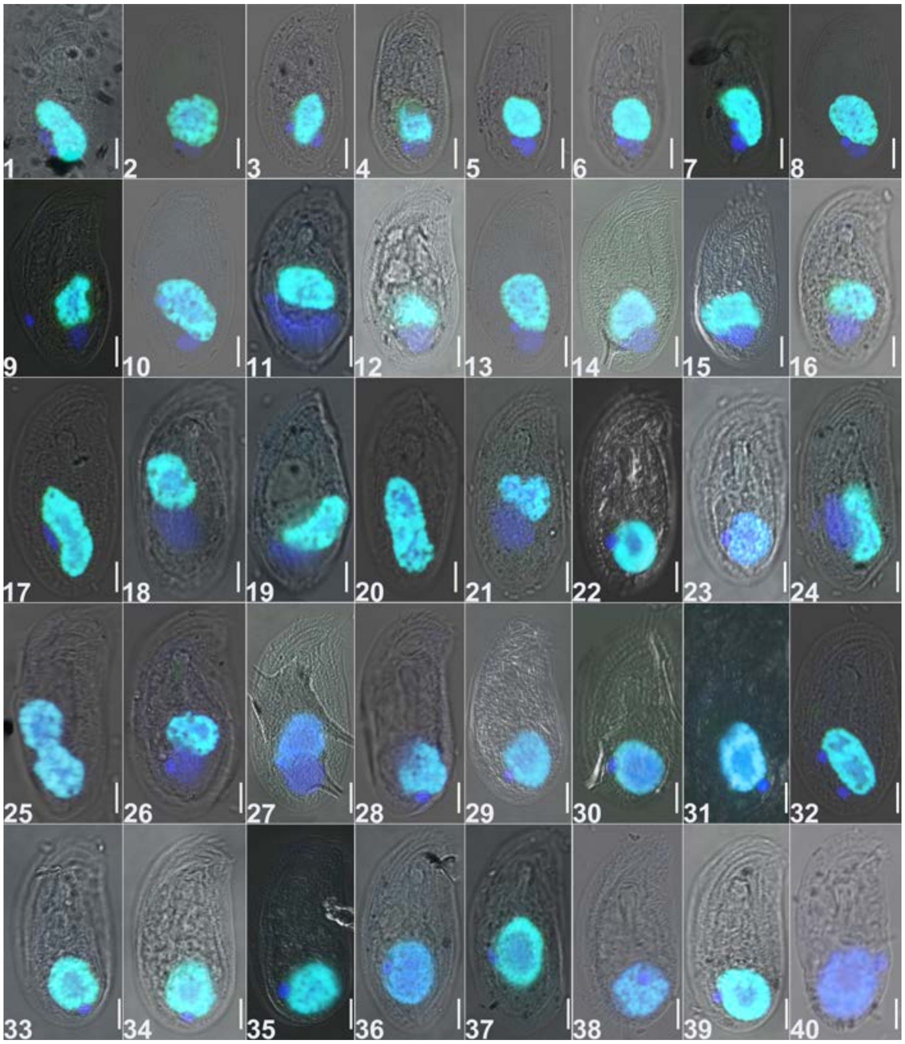

1
2
3
4
5
6
7
8
9
10
11
12
13
14
15
16
17
18
19
20
21
22
23
24
25
26
27
28
29
30
31
32
33
34
35
36
37
38
39
40

Figure S6:

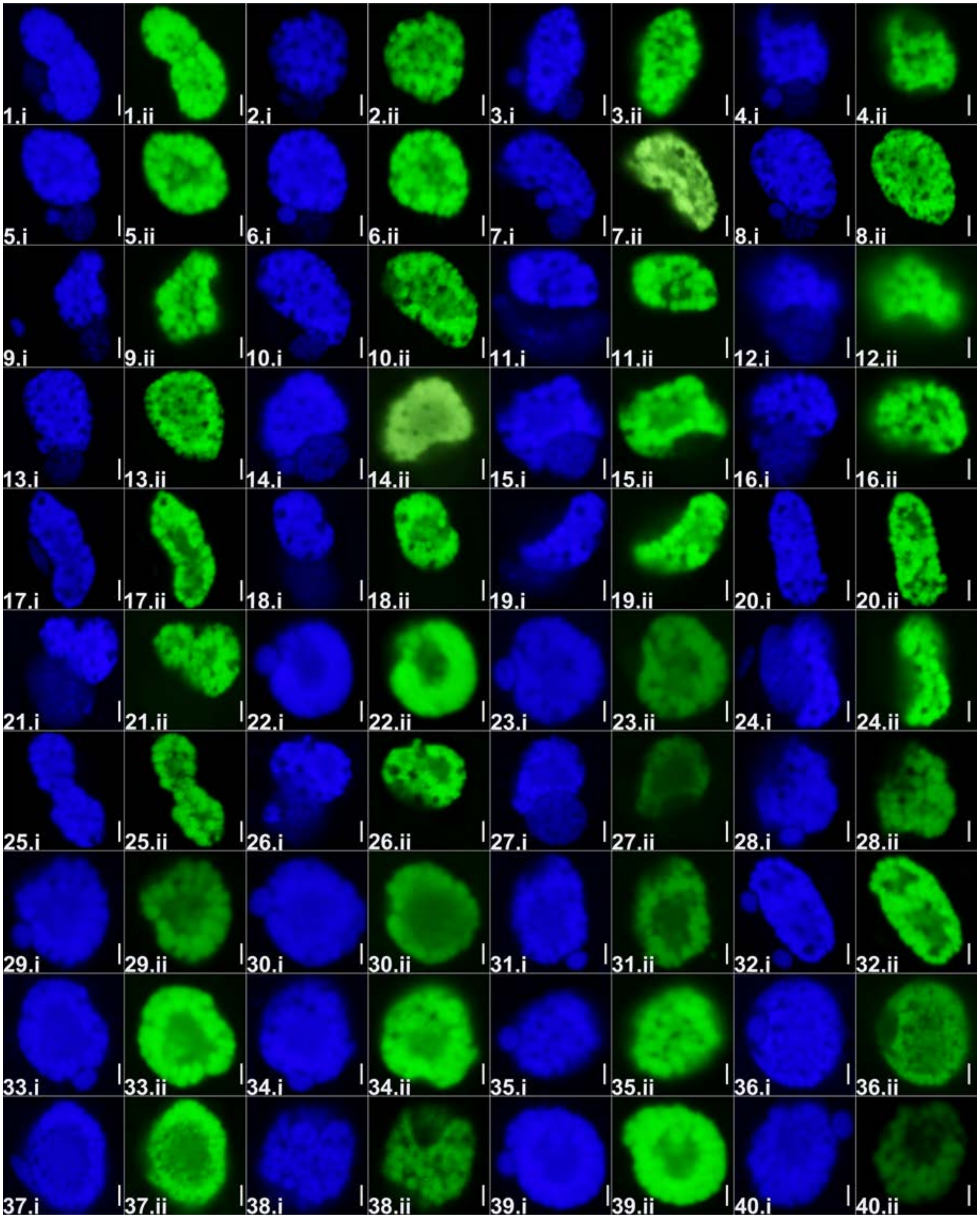
1.i
1.ii
2.i
2.ii
3.i
3.ii
4.i
4.ii
5.i
5.ii
6.i
6.ii
7.i
7.ii
8.i
8.ii
9.i
9.ii
10.i
10.ii
11.i
11.ii
12.i
12.ii
13.i
13.ii
14.i
14.ii
15.i
15.ii
16.i
16.ii
17.i
17.ii
18.i
18.ii
19.i
19.ii
20.i
20.ii
21.i
21.ii
22.i
22.ii
23.i
23.ii
24.i
24.ii
25.i
25.ii
26.i
26.ii
27.i
27.ii
28.i
28.ii
29.i
29.ii
30.i
30.ii
31.i
31.ii
32.i
32.ii
33.i
33.ii
34.i
34.ii
35.i
35.ii
36.i
36.ii
37.i
37.ii
38.i
38.ii
39.i
39.ii
40.i
40.ii

Figure S7:

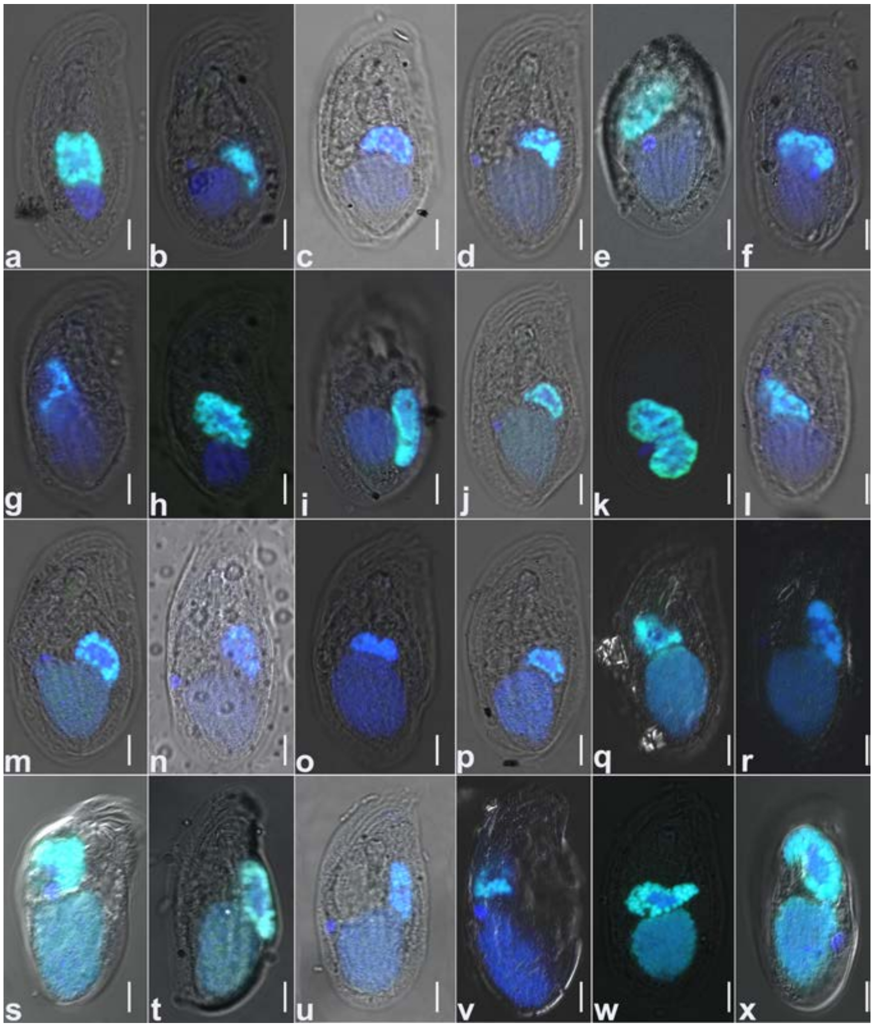

Figure S8:

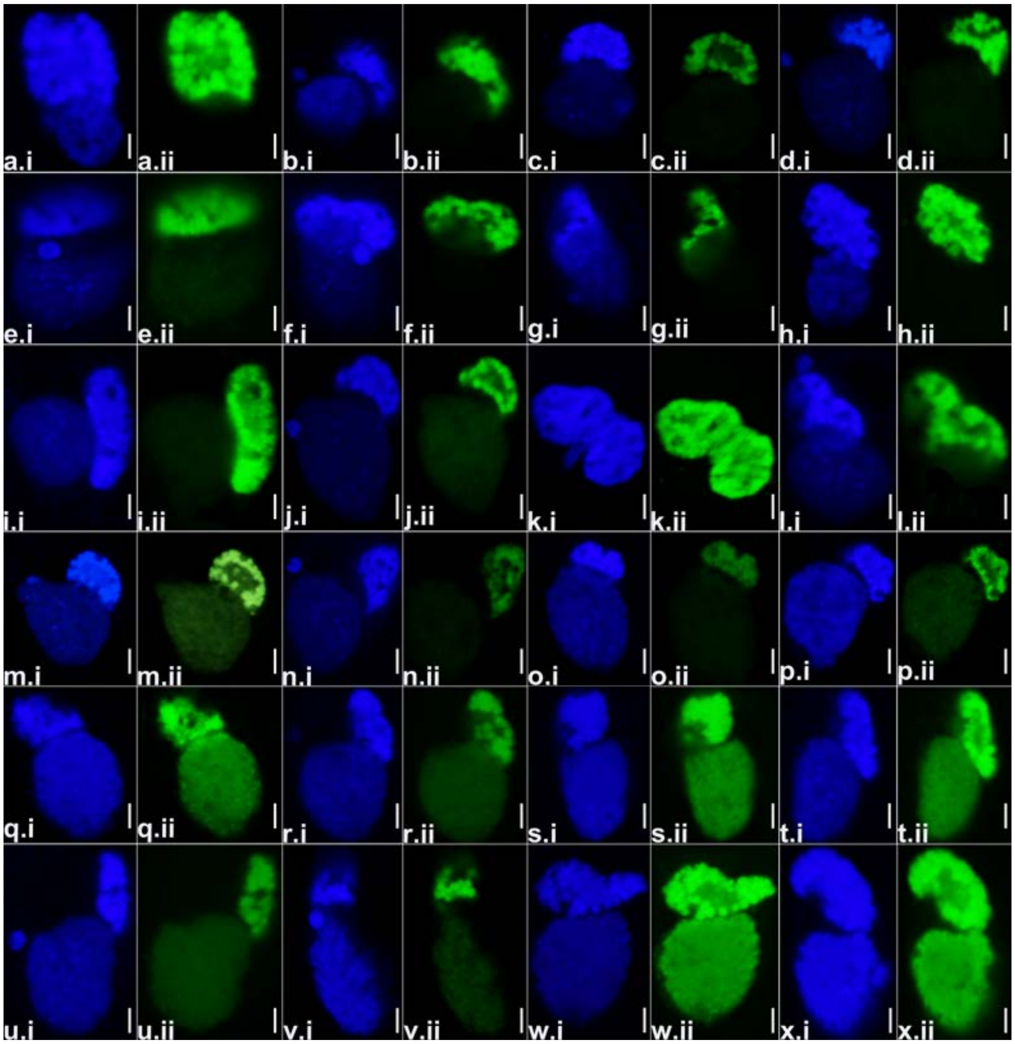
a.i
a.ii
b.i
b.ii
c.i
c.ii
d.i
d.ii
e.i
e.ii
f.i
f.ii
g.i
g.ii
h.i
h.ii
i.i
i.ii
j.i
j.ii
k.i
k.ii
l.i
l.ii
m.i
m.ii
n.i
n.ii
o.i
o.ii
p.i
p.ii
q.i
q.ii
r.i
r.ii
s.i
s.ii
t.i
t.ii
u.i
u.ii
v.i
v.ii
w.i
w.ii
x.i
x.ii

Figure S9:

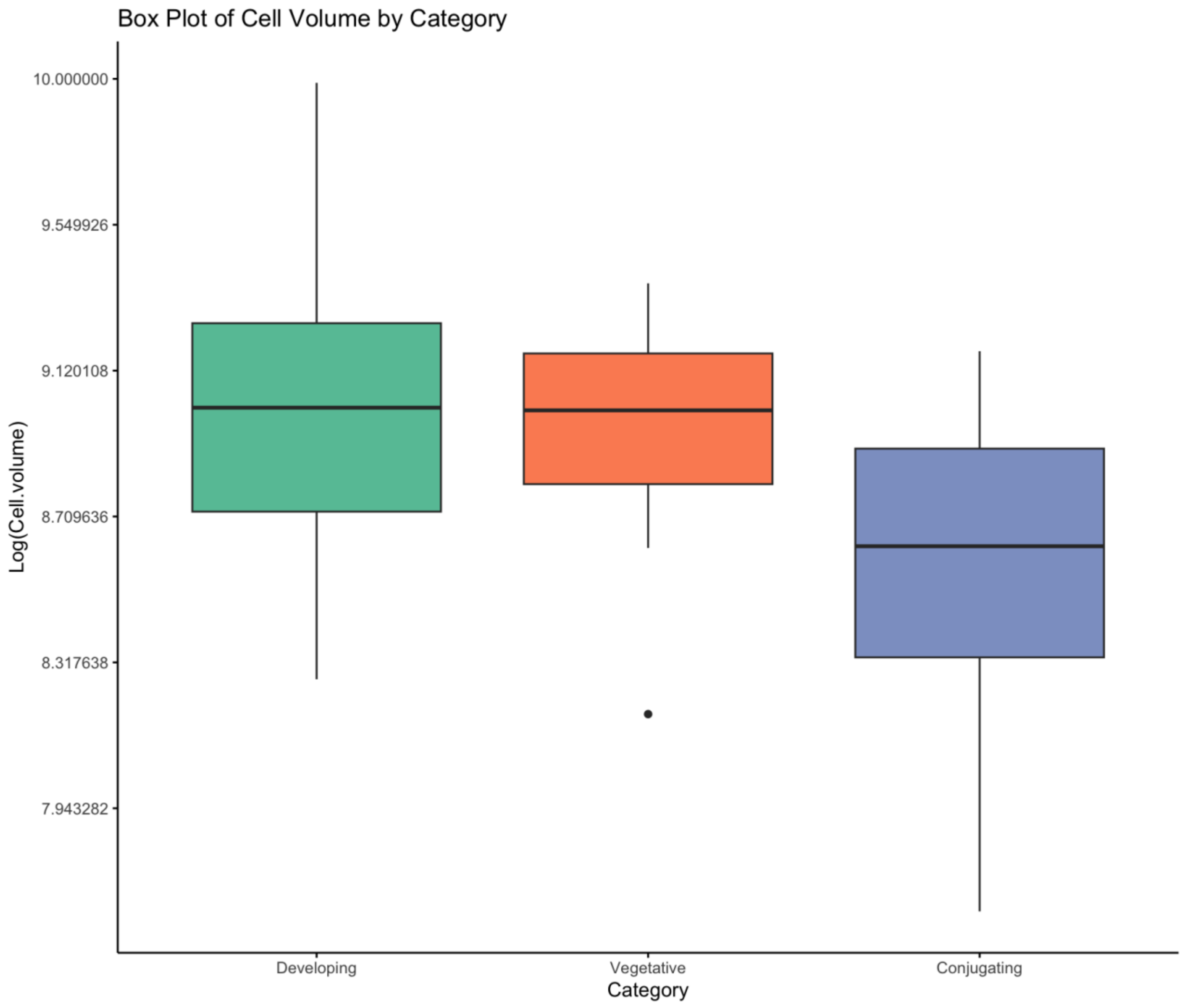

Box Plot of Cell Volume by Category
10.000000
9.549926
9.120108
8.709636
8.317638
7.943282
Log(Cell.volume)
Developing
Vegetative
Conjugating
Category

Figure S10:

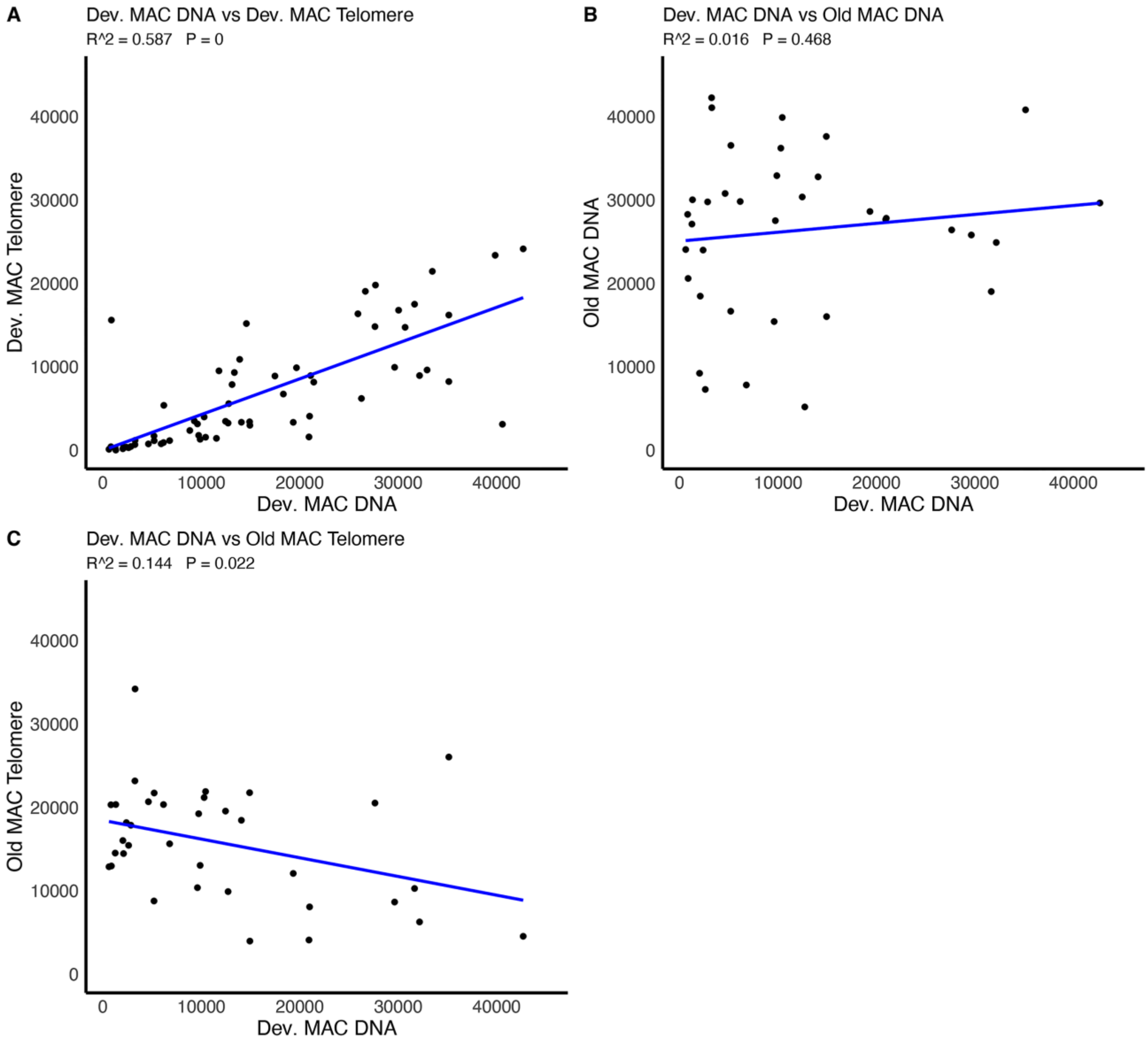
A
Dev. MAC DNA vs Dev. MAC Telomere
R^2 = 0.587   P = 0
Dev. MAC Telomere
Dev. MAC DNA
B
Dev. MAC DNA vs Old MAC DNA
R^2 = 0.016   P = 0.468
Old MAC DNA
Dev. MAC DNA
C
Dev. MAC DNA vs Old MAC Telomere
R^2 = 0.144   P = 0.022
Old MAC Telomere
Dev. MAC DNA
0
10000
20000
30000
40000

Figure S11:

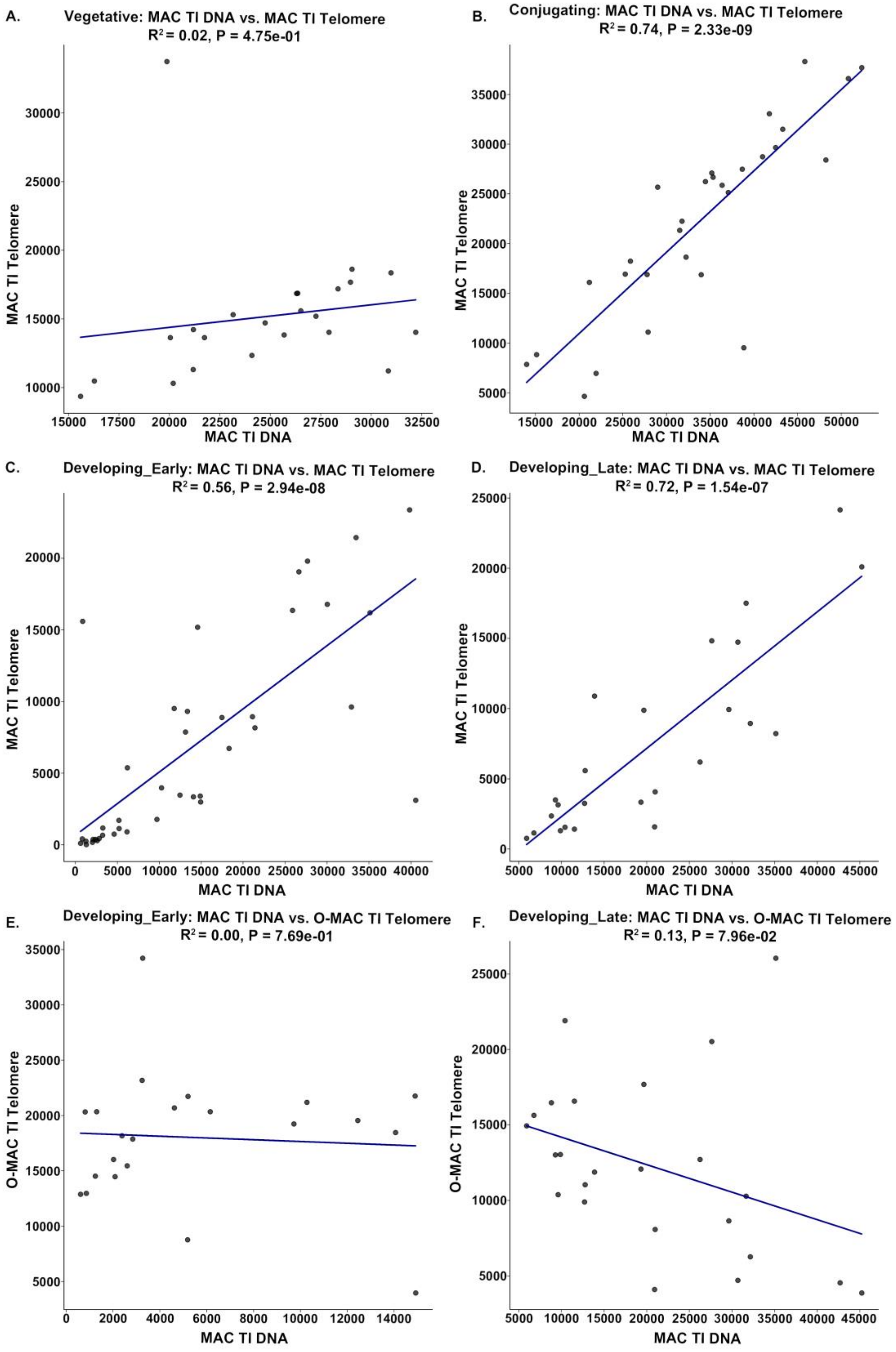
A.
Vegetative: MAC TI DNA vs. MAC TI Telomere
R2 = 0.02, P = 4.75e-01
MAC TI Telomere
MAC TI DNA
B.
Conjugating: MAC TI DNA vs. MAC TI Telomere
R2 = 0.74, P = 2.33e-09
MAC TI Telomere
MAC TI DNA
C.
Developing_Early: MAC TI DNA vs. MAC TI Telomere
R2 = 0.56, P = 2.94e-08
MAC TI Telomere
MAC TI DNA
D.
Developing_Late: MAC TI DNA vs. MAC TI Telomere
R2 = 0.72, P = 1.54e-07
MAC TI Telomere
MAC TI DNA
E.
Developing_Early: MAC TI DNA vs. O-MAC TI Telomere
R2 = 0.00, P = 7.69e-01
O-MAC TI Telomere
MAC TI DNA
F.
Developing_Late: MAC TI DNA vs. O-MAC TI Telomere
R2 = 0.13, P = 7.96e-02
O-MAC TI Telomere
MAC TI DNA

Figure S12:

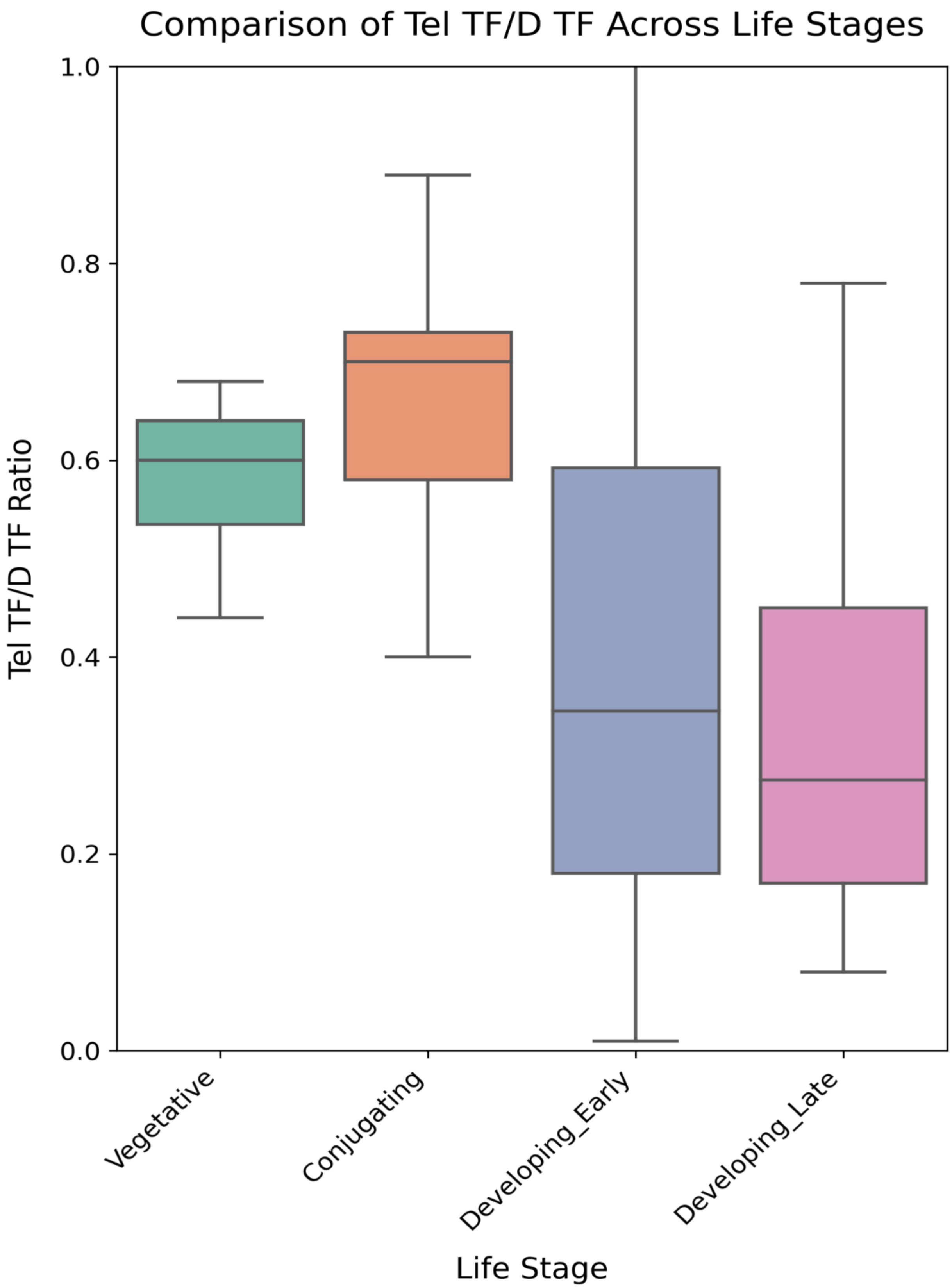

Comparison of Tel TF/D TF Across Life Stages
Tel TF/D TF Ratio
1.0
0.8
0.6
0.4
0.2
0.0
Vegetative
Conjugating
Developing_Early
Developing_Late
Life Stage
